# Thai-STEADI Program: Roles of Community Health Workers and Care Managers to Prevent Falls in LMICs

**DOI:** 10.1093/geroni/igaf122.983

**Published:** 2025-12-31

**Authors:** Ladda Thiamwong, Rui Xie, Boon Ng, Sirinart Tongsiri, Jom Suwanno

**Affiliations:** University of Central Florida, Orlando, Florida, United States; University of Central Florida, Orlando, Florida, United States; University of Central Florida, Orlando, Florida, United States; Mahasarakham University, Maha Sarakham, Maha Sarakham, Thailand; Walailak University, Tasala, Nakhon Si Thammarat, Thailand

## Abstract

One-third to half of older adults in low and middle-income countries (LMICs), including Thailand, have falls, fear of falling, and physical inactivity. There is a lack of pragmatic and cost-effective multi-factorial fall prevention interventions embedded within primary care settings led by trusted and trained local health personnel to ensure interventions reach older adults who need them the most and enhance the intervention’s sustainability. This leaves clinical practice bereft of knowledge to prevent falls in LMICs. Our team worked closely with community partners in Thailand in 4 steps of cultural adaptation and pilot-testing the CDC’s Stopping Elderly Accidents, Deaths, and Injuries (STEADI) with input from Thai older adults, community health workers, and care managers. Our pilot study (n = 27) indicated that Thai-STEADI delivered by community health workers and care managers is feasible, appropriate, and acceptable for low-income older adults with limited health care access. The Thai-STEADI group had a significant reduction in fall risk from pre-intervention (M = 11.60, SD = 1.52) to post-intervention (M = 10.80, SD = 1.64) but not the control group. Thai-STEADI also group improved dynamic balance and the mean of the Timed-Up and Go test scores decreased from 20.24 (SD = 23.58) to 19.60 (SD = 23.31), but the decrease was not statistically significant. Fear of falling was reduced in the Thai-STEADI group from 100% to 80%, whereas 100% of the control group still had a fear of falling. Using a combination of community health workers and care managers with increasing stakeholder influence is one of the most vital strategies to prevent falls at a system level in LMICs.

